# A Closer Look: Crafting Your Community Advisory Board (CAB): Important Dont’s When Developing a CAB as Part of Your Research

**DOI:** 10.1002/pon.70387

**Published:** 2026-01

**Authors:** Leah E. Walsh, Shena Gazaway, Joi Miner, Ronit Elk

**Affiliations:** 1 Brookdale Department of Geriatrics and Palliative Medicine, Icahn School of Medicine at Mount Sinai, New York, New York, USA; 2 School of Nursing, The University of Alabama at Birmingham, Birmingham, Alabama, USA; 3 Community Member; 4 Department of Medicine, Division of Gerontology, Geriatrics, and Palliative Care, The University of Alabama at Birmingham, Birmingham, Alabama, USA

As researchers, we think of the things we need to do: we need to call the study participant, analyze that subset of the data, address manuscript revisions, and meet to strategize the next grant application. But how often do we reflect on what we should *not* do — approaches we should steer away from, mistakes we do not want to repeat, and those factors that would characterize “bad practice”? When creating a community advisory board (CAB) for a research project, we, understandably, focus on the things we need to do to foster community. However, it is equally important to consider what we should *avoid doing* as we seek to ensure our CAB is co-created and supported as effective and rooted in community values as possible.

## 1 | Don’t Misalign Your Potential CAB Members With Your Issue and Population

A strong conceptualization of your study’s purpose is necessary to guide the creation of your CAB. Even more, the community population that you are hoping to study needs to be aligned and in agreement with the importance and relevance of the issue you are studying to the greater needs of the population. For example, if you are studying patients with a particular cancer diagnosis or experience of a specific treatment, you need to ensure that potential CAB members have some type of experience with or connection to that diagnosis or treatment and can speak to the potential challenges you are hoping to better understand. You need members who can serve as a connection between the community and the issue and speak from that perspective. If community members don’t see how this research study will help to address a challenge that they are experiencing, such as adherence to a specific treatment regimen, or improving coping with cancer-related mental health challenges, they won’t be interested in joining. Utilize connections to the community to understand how they view the importance and/or role of your research.

Remember that building community relationships takes time. Start by connecting with a community leader, learning about their perspective of challenges in that community, and having them “open doors” for you when trust is established. Talking to those directly impacted by the issue is necessary to understand what they need and whether focusing on this issue to jointly create a solution is of importance to them. This will also foster inclusion and joint decision-making. Once you have buy-in from community groups, you can identify the types of individuals needed to contribute to a well-aligned CAB.

## 2 | Don’t Forget About CAB Dynamics

A successful CAB fosters a warm, inclusive, and curious environment where robust representation across backgrounds exists and where trust builds and grows as you continue to work together. Ensure that you don’t have just one individual from a different background (e.g., race, ethnicity, sexual or gender identity, age, experience); this may lead to a power imbalance as certain individuals struggle to have their voices represented when standing alone. While strength comes from multiple perspectives, so, too, does having balance across the number of voices representing different backgrounds. When finding a balance in CAB members is difficult, look for the candidates who are the best representative of their experience and ensure that they understand they may offer solitary, personal feedback, but may also be able to give insight on the collective group as well. CAB members should want to hear and respect all voices at the table. Researchers can work to ensure that CAB members who often speak up are appreciated for their contributions and can intentionally create space in the meeting to invite other members to share their perspectives. Humility should be at the forefront of each interaction between researchers and CAB members, where researchers are open to being corrected or challenged by CAB members as experts in their experience. A healthy CAB mindset is motivated by the idea that *no one person knows everything, but we can figure it out together.* Tools and resources exist for researchers to think about their CAB representation to increase awareness of whether particular voices are missing (see Table 1).

As a researcher, you are a part of the CAB dynamic and can shape and set the tone for interactions. Even more, CAB dynamics can be enhanced by getting to know members more deeply. Some might have skills that could aid your research in both conventional and unconventional ways! Sharing aspects of your own identity (e.g., your racial or cultural background and connection to the issue at hand) while maintaining boundaries can help break down the wall between researchers and community members.

## 3 | Don’t Default and Reuse the Same CAB Every Time—Stay Anchored to Your Study’s Purpose

You might already have an established CAB within your center, department, or research team. Perhaps you have established connections with patients with specific brain cancer diagnoses, or with healthcare providers at a particular center within your cancer hospital. But do those members have experience with the issue you are studying specifically?

There may be times when a CAB is program-based, and engaging with the same CAB across studies may serve to ensure that, as thoughts pivot, actions pivot. Other times, even when it is time-effective to pull from existing resources, defaulting to previous CABs may not be the best to maintain study alignment. CAB member selection needs to be directly related to the issue at hand. You need members who can be the connection between the community and the issue and speak from that perspective. Downstream, a CAB that lacks a clear resonance to the study at hand can negatively impact the integrity of the research and do a disservice to the population you are trying to support.

Think about what groups are impacted by the issue you are studying.

- In addition to patients, consider including patients’ family or friend caregivers who often shoulder immense responsibilities that impact their own mental and physical health.
- Community leaders can provide recommendations of members who may be great fits for your CAB.
- CABs can also extend to organizations that have invested commitments to that issue, such as foundations and associations that provide education, financial resources, and other support critical to living with that disease or condition. Organizations may also have established connections with people interested in participating in a CAB.
- Colleagues in clinical settings may know patients who experience the issue of study who may be interested in contributing to a solution to this problem.

## 4 | Don’t Reach out to Potential CAB Members Until You’ve Thought About Responsibilities—Yours and Theirs

Transparency from the first interaction with potential CAB members is paramount. Document all responsibilities that you have to CAB members, and that they possess as a CAB member [1]. These roles and boundaries should be mutually agreed upon by all CAB members and stakeholders involved in the research. Ask members what level of commitment they are willing and able to have—how much time can they commit? Will that change? If patients are on your CAB, consider how participation may interact with their medical care and appointments. If caregivers are on your CAB, consider how their responsibilities to the patient and other family, occupational, or personal roles may interact with CAB involvement. Provide options for engagement: some may want to be involved at the beginning of the study to frame the work, and others may be interested in participating in the dissemination of results. Responsibilities and all communication with CAB members should be communicated in lay language.

Are you willing to modify aspects of your research if the CAB doesn’t think it will be effective? How will you address conflicting suggestions made by CAB members? While these decisions may not be made while constructing your CAB, you should consider your responsibilities to those involved in the research and examine how you will lead in a respectful and transparent manner. Part of your responsibilities as a researcher is to thoughtfully and critically consider all suggestions and recommendations made by the CAB, grounded in honesty and transparency, to build a strong relationship with the CAB.

## 5 | Don’t Approach CAB Members Through Ineffective Strategies

There are many ineffective ways to find members for your CAB. Don’t go to a community that you are not from, or don’t have established connections with, and knock on doors to see if people are interested in your study. This is an immediate red flag for most underserved communities and will prove to be a hindrance not only to your study but to future studies as well. There is a great deal of mistrust within many communities toward outsiders, particularly in relation to medical research. This mistrust is real and rooted in previous studies that took advantage of communities, breeding significant and pervasive mistrust that persists to this day [2–4]. While flyers and social media are great tools for recruitment, they may not be effective at attracting people who have the direct experience necessary for participation.

## 6 | Don’t Skip Reflecting on Your Own Identities With Respect to the Issue of Study

Have you thought about what it means to work with the groups of people who hold identities you may not share? What about what it means to be a researcher and how this identity impacts your perspective? Does your researcher or academic identity intersect with others that you hold? Each member of the study team, regardless of their role, should reflect on what it means to be, in some way, connected to community work [5]. As an academic research team leader and researcher, it is your responsibility to promote frequent reflection on how the CAB guides your research processes. You set an example for the study staff to reflect on privilege. Exercises in power can help to facilitate this reflection [6]. Examples of such exercises to address power differentials are Implicit Bias Training (e.g., identifying unconscious judgments we hold based on stereotypes) [7, 8], Structural Competency (e.g., recognizing the systemic, institutional, and policy barriers that cause social and health inequities, and keeping this knowledge at the forefront of your actions) [9], and Positionality (e.g., conscious awareness of the researcher’s own identities and lived experiences influences their perspective in research, and how their status has been given based on education, their institution, or their own demographic identity) [5, 6, 10]. These types of reflections should not be static, but recurring and evolving conversations with team members to bring biases to the surface and examine how they may or may not impact the issue of study and the interactions with and roles of the CAB.

Though crafting your CAB is just one step in the ongoing process of engaging community members in your research, it sets the tone for how you think about, and how you respect, the positions of your CAB members. For many, a cancer diagnosis can be seen as a “death sentence” due to a lack of education or misunderstanding about treatment options. CABs in psychosocial oncology require a safe space for vulnerabilities to be expressed, acknowledged, and appreciated. The co-existence of grieving one’s life they thought they would have prior to cancer and hope for the future is possible if CABs are constructed with sensitivity. A strong and vibrant CAB that is created through self-guided reflection and is designed to nurture respect and humility can foster richer studies with more impactful and equitable findings and results for the communities where we live, love, work, and play—and the communities where we grow sick, die, and grieve. Creating research that unites community members and researchers can shift the narrative from one filled with apprehension to one of trust and positive inclusivity.

## Figures and Tables

**TABLE 1 |:** Closing out: key takeaways about crafting a CAB.

1. Be transparent from day one. Think about and write out how often the CAB will meet, where, and at what time, how long meetings will last, how they will be paid, and, perhaps most importantly, whether food will be present (particularly for meetings around breakfast or dinner). If your CAB includes patients with cancer on treatments that lead to fatigue, nausea, or other side effects, or compromise their immunity, offer participation in sensitive formats (i.e., virtual meetings).
2. Prior to starting your CAB, make a table. In column 1, list all the types of representation you need on the CAB (e.g., community leaders, patients with a particular cancer type, stage, or treatment experience, caregivers, healthcare providers who care about this issue) and in column 2, insert the people you have recruited for this “category” and identify the ways that they are aligned with the study goal or issue of study.
3. Exercises in power can promote reflection across all members of the study team as to why community work is an important pillar of your study. Some examples are: Implicit bias training, structural competency, positionality.
4. Throughout each step of creating your CAB, remember that this group should foster a safe space for individuals motivated to improve the current issue. This can facilitate a long-standing, trustworthy relationship with CAB members.
5. There are many great trainings in community-based participatory research. This type of training is imperative to fostering a collaborative and engaged approach across all aspects of your study. Consider some of the following options: https://www.urmc.rochester.edu/clinical-translational-science-institute/education/cbpr-training https://www.uab.edu/cores/ircp/mmr-cbpr-qr/resources

## Data Availability

The authors have nothing to report.
